# Gene expression profiles and bioinformatics analysis of insulin‐like growth factor‐1 promotion of osteogenic differentiation

**DOI:** 10.1002/mgg3.921

**Published:** 2019-08-16

**Authors:** Yashuai Yuan, Ruimeng Duan, Baolin Wu, Wei Huang, Xiuzhi Zhang, Mingjia Qu, Tao Liu, Xiaobing Yu

**Affiliations:** ^1^ Department of Orthopaedics Affiliated Zhongshan Hospital of Dalian University Dalian China

**Keywords:** IGF‐1, MC3T3‐E1 cells, Proliferation, RNA‐seq

## Abstract

**Background:**

Insulin‐like growth factor‐1 (IGF‐1) promotes osteoblast differentiation and mineralization. The objective of this study was to investigate the effects of IGF‐1 on proliferation, mineralization, alkaline phosphatase (ALP) synthesis, and gene expression of osteoblast differentiation in MC3T3‐E1 osteoblasts cells, and to explore gene expression profiling differential genes.

**Methods:**

MC3T3‐E1 osteoblasts cells were cultured in medium with or without IGF‐1. The ALP assay was employed to determine the osteoblast mineralization, and Alizarin red S to stain for calcium deposits, which were the indicators of mature osteocytes. The living cell number was assessed by the Cell Counting Kit‐8 method. RNA‐seq analysis was applied to identify genes that were differentially expressed in with or without IGF‐1 as well as genes that varied between these two groups. The expression of osteogenic marker genes was determined by quantitative real‐time polymerase chain reaction (qRT‐PCR) and western blot analysis.

**Result:**

The cell number of osteoblasts exposed to IGF‐1 at 200 μg/L significantly increased compared with the control group. The ALP activity in IGF‐1‐treated cells was higher than that in the control group. IGF‐1 can increase ALP synthesis in osteoblasts in vitro. RNA‐seq analysis showed that 677 triggered differentially expressed genes by IGF, of which 383 genes were downregulated and 294 genes were upregulated. Gene ontology (GO) analysis showed that IGF‐1 caused a significant change in gene expression patterns.

**Conclusions:**

This result suggested that IGF‐1 could probably promote the synthesis of organic matrix and mineralize action of bone. Osteogenic‐related genes (*DMP1, PHEX, SOST, BMP2, RUNX2, OPN,* and *OCN*) were significantly upregulated both in GO analysis and in pathway analysis to perform qRT‐PCR. Western blot analysis demonstrated that the Notch pathway was highly upregulated in MC3T3‐E1 cells.

## Graphical abstract

This article suggested that insulin‐like growth factor‐1 could probably promote the synthesis of organic matrix and mineralize action of bone. Osteogenic‐related genes (*DMP1*, *PHEX*, *SOST*, *BMP2*, *RUNX2*, *OPN*, and *OCN*) were significantly upregulated both in gene ontology analysis and pathway analysis to perform quantitative real‐time polymerase chain reaction. Western blot analysis demonstrated that the Notch pathway was highly upregulated in MC3T3‐E1 cells.

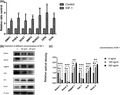

## 1 INTRODUCTION

Osteoblasts are the major cellular components of bone and account for approximately 90%–95% of all bone cells (Féron & Mauprivez, 2016). Osteoblasts are the mainstay of bone formation and skeletal development and growth. For cells, the main function in bone formation is synthesis and to secrete collagen to form the bone matrix and release calcium ions. Matrix calcification completes bone formation, and osteoblasts are differentiated from mesenchymal stem cells (MSCs) (Partap, Plunkett, Kelly, & O'Brien, 2010). The signal transduction mechanism of MSC differentiation into osteoblasts has been studied extensively, but the mechanism of osteoblast cell transformation to bone cells is still being studied (Bonewald, 2017). Osteocytes secrete large amounts of insulin‐like growth factor‐1 (IGF‐1) in bone. Although IGF‐1 is produced locally by other bone cells, such as osteoblasts and chondrocytes, it has been shown to play an important regulatory role in bone turnover and developmental bone growth (Sheng, Lau, & Baylink, 2014). IGF‐1 is a growth‐promoting cytokine that plays an important role in development, metabolism, and growth (Qiu et al., 2018). IGF‐1 promotes osteoblast differentiation and mineralization, which is likely to be an important inducer of bone formation, callus healing, and fracture healing in vivo (Guo et al., 2017).

IGF promotes cell proliferation and matrix synthesis, has a high content in the fracture site during early healing, and plays a promoting role in the formation of new bone (Koh et al., 2011). Xing et al., (2015) reported that the use of mesenchymal stromal cells transfected with IGF‐1 could promote fracture healing in diabetic animals. The functional role of osteocyte‐derived IGF‐1 in bone and mineral metabolism has not been investigated and remains unclear. To further clarify the effect of IGF‐1 on the proliferation and differentiation of mouse osteoblasts and to determine which signal transduction pathway plays a role in this process, we use IGF‐1 to act on MC3T3‐E1 osteoblasts to investigate IGF‐1. It is known that *DMP1* (OMIM:600980), *PHEX* (OMIM:300550), *SOST* (605740), *BMP2* (OMIM:112261), and *RUNX2* (OMIM:600211) are important genes in the process of osteogenic differentiation. We examined the effects on the proliferation and differentiation of osteoblasts and the expression of signaling pathways during this process. Through gene sequencing, we investigated the gene expression changes of MC3T3‐E1 osteoblasts induced by IGF, the difference of gene ontology (GO) analysis, and the pathways of different gene expression.

## 2 MATERIALS AND METHODS

### 2.1 Ethical compliance

All our study was approved by Ethics Committee of Affiliated Zhongshan Hospital of Dalian University.

### 2.2 Cell culture

We grew MC3T3‐E1 mouse preosteoblasts (American Type Culture Collection) in Dulbecco's modified Eagle medium (HyClone) supplemented with 10% fetal bovine serum, 100 IU/ml penicillin, and 100 µg/ml streptomycin (Invitrogen) in an incubator at 37°C with 5% CO_2_.

### 2.3 Alkaline phosphatase staining

The alkaline phosphatase (ALP) assay was employed to determine the osteoblast mineralization of MC3T3‐E1 osteoblast cells treated with various concentrations of IGF‐1 (0, 10, 50, 100, and 200 µg/L). We seeded the cells in six‐well plates at a density of 2 × 10^4^ cells per well and treated with various concentrations of IGF‐1 (0, 10, 50, 100, and 200 µg/L). After 14 days of differentiation, we washed the osteoblasts twice with phosphate‐buffered saline (PBS), fixed with 4% paraformaldehyde for 10 min, rinsed them with deionized water, and stained with a BCIP (5‐bromo‐4‐chloro‐3‐indolyl‐phosphate)/NBT (nitro blue tetrazolium) ALP color development kit (Beyotime, Institute of Technology) for 1 hr while they were protected from direct light according to the manufacturer's instructions. Then, we obtained the images using a phase‐contrast microscope equipped with a digital camera; we regarded the areas that were stained purple as positive.

### 2.4 Alizarin red S staining

We used Alizarin red S to stain for calcium deposits, which were indicators of mature osteocytes. We seeded osteoblast cells in six‐well plates at a density of 2 × 10^4^ cells per well and treated with various concentrations of IGF‐1 (0, 10, 50, 100, and 200 µg/L). We then added osteoblasts cultured in vitro into the medium. On the 14th day of differentiation, we fixed the osteoblasts in 4% paraformaldehyde phosphate buffer for 10 min at room temperature. We washed the cells with ddH_2_O and stained with 1% (wt/vol) alizarin red at pH 4.4 for 40 min at room temperature. Then, we rinsed the samples twice with ddH_2_O. We captured the images of stained cells using a phase‐contrast microscope equipped with a digital camera. Six independent experiments were quantified after capturing images using a microscope (×200 magnification) equipped with a Tucsen ISH 500 CCD camera.

### 2.5 Cytotoxicity test

We seeded MC3T3‐E1 mouse preosteoblasts in 96‐well plates at a density of 5 × 10^3^ cells per well, treated them with various concentrations of IGF‐1 (0, 10, 50, 100, and 200 µg/L), and cultured them in an incubator for 1, 3, 5, and 7 days. We used a Cell Counting Kit‐8 (Dojindo, Molecular Technologies) to evaluate the cytotoxic effect of IGF‐1. Briefly, we added medium (1 ml) containing 100 µl of CCK‐8 to each precultured well, and incubated the plates for 2 hr at 37°C. We determined the absorbance at a wavelength of 450 nm using a microplate reader (BioTek, Instruments, EPOCH2). The values were then tabulated.

### 2.6 RNA‐seq

We isolated the total RNA using Trizol reagent (Gibco, 15596–018, Thermo Fisher Scientific) according to the manufacturer's protocol. We prepared whole RNA‐seq libraries and deep sequencing by the AnnoroadGene Technology Corporation. We measured RNA integrity number and the concentration using a 2100 RNA Nano 6000 Assay Kit (Agilent Technologies). We enriched the mRNA with Oligo (dT) mRNA magnetic beads. We prepared RNA‐seq libraries using 6‐bp random primers and sequenced the libraries on the IlluminaHiSeq X‐Ten with 150‐bp paired‐end reads. We mapped RNA‐seq reads to the mouse genome (mm10) using TopHat v2.0.12. We used reads per kilobase million mapped reads to quantitatively estimate gene expression values. We used the final set of the genes for differential expression using DEGseq to compare genes that were upregulated and downregulated in MC3T3‐E1osteoblast cells using hypergeometric distribution.

### 2.7 Western blot analysis

We used RIPA buffer containing a protease inhibitor (20 mM Tris [pH 7.5], 150 mM NaCl, 1% Nonidet P‐40, 0.5% sodium deoxycholate, 1 mM ethylenediaminetetraacetic acid, and 0.1% sodium lauryl sulfate [SDS]). We extracted total protein lysate cocktail (Sigma Aldrich). We measured protein concentration using the Bradford method (Bio‐Rad Laboratories) and separated 15–30 μg of each sample by 10%–12% SDS‐polyacrylamide gel electrophoresis and blotted to a nitrocellulose membrane (Hybond)—ECL (Amersham Biosciences). Nonspecific‐binding sites were blocked by incubation with 5% skim milk powder in Tris‐buffered saline–0.1% Tween‐20. Mouse β‐actin monoclonal antibody (working concentration: 1:1,000), mouse Notch1 monoclonal antibody (working concentration: 1:1,000), goat Notch2 polyclonal antibody (working concentration: 1:500), goat Jagged1 polyclonal antibody (working concentration: 1:500), goat Runx2 polyclonal antibody (working concentration: 1:500), rabbit source Hes1 polyclonal antibody (working concentration: 1:1,000), goat macrophage colony‐stimulating factor (MCSF) polyclonal antibody (working concentration: 1:500), goat ALP polyclonal antibody (working concentration: 1:1,000), rabbit‐derived RANK ligand (RANKL) polyclonal antibody (working concentration: 1:500), mouse source osteoprotegerin (OPG) polyclonal antibody (working concentration: 1:500) were incubated for 1 hr at room temperature, and then the membrane was washed three times with PBS with Tween 20 (PBST) for 10 min each time. Then, the corresponding secondary antibody (working concentration 1:15,000) was incubated at room temperature for 1 hr, and then washed with PBST three times for 10 min each time to detect the expression of various proteins using BIO‐RADChemiDocTM XRS+ imaging system and developer.

### 2.8 Statistical analysis

We carried out statistical analyses using Student's *t*‐test to compare the control and treated groups, and paired the data using the SPSS version 20.0 software. We expressed the data as means ± *SE*. We used a one‐way analysis of variance, followed by Tukey tests for multiple comparisons wherever appropriate. We used *p* < .05 to indicate statistical significance. We performed statistical analysis using the software package GraphPad Prism (Prism 5.01; GraphPad Software).

## 3 RESULTS

### 3.1 Effect of IGF‐1 on osteoblast ALP activity

**Figure 1:**
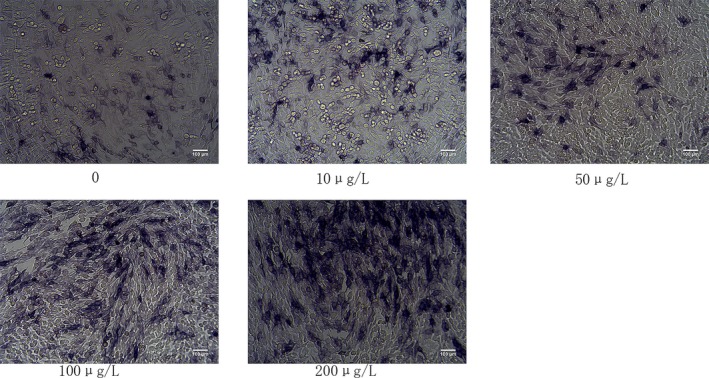
Alkaline phosphatase (ALP) staining for early mineralization activity induced by 14 days of IGF‐1 treatment in MC3T3‐E1 cells. The cells were cultured in six‐well plates for 14 days with 0, 10, 50, 100, and 200 μg/L. ALP staining showed the expression and enzymatic activity of the phosphatase in MC3T3‐E1 cells. Early phase mineralization was measured using ALP staining

The effect of IGF‐1 on osteoblast ALP activity often is used as an indicator of osteoblast function. In this study, we determined whether IGF‐1 treatment of MC3T3‐E1 cells stimulated ALP activity, which accelerated the mineralization process at an early stage. We stained ALP‐positive human osteoblasts with ALP staining, which reacted with BCIP/NBT to render the cells purple. ALP staining showed that IGF‐1 treatment increased ALP activity in MC3T3‐E1 cells. We observed that 10, 50, 100, and 200 μg/L of IGF‐1 increased the number of ALP‐positive cells after 14 days of culture in MC3T3‐E1 cells. Among them, the 200 μg/group of ALP activity increased most obviously (Figure 1).

### 3.2 Effect of IGF‐1 on mineralization of MC3T3‐E1 cells

**Figure 2:**
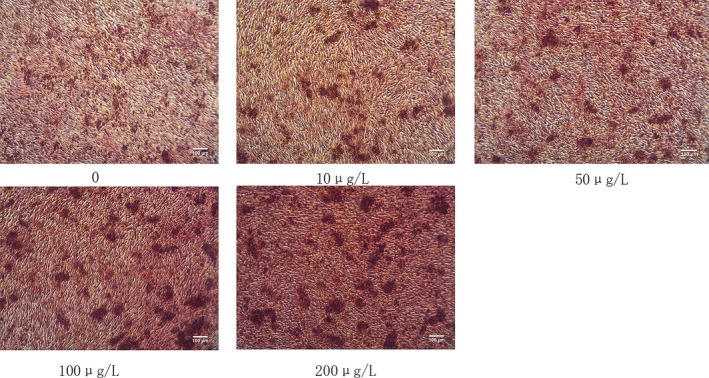
Alizarin red S staining for calcium deposits (i.e., bone nodules) induced by 14 days of IGF‐1 treatment in MC3T3‐E1 cells. Cells were cultured in six‐well plates for 14 days with 0, 10, 50, 100, and 200 μg/L. Extracellular matrix Ca^2+^ deposits indicate that matrix mineralization was detected using alizarin red S dye, which binds Ca^2+^. IGF, insulin‐like growth factor‐1

Extracellular matrix mineralization is a major component of bone formation. In this study, we determined whether IGF‐1 treatment of MC3T3‐E1 cells stimulates matrix mineralization, thereby increasing anabolic activity during bone metabolism. We stained the extracellular matrix Ca^2+^ deposits used for mineralized nodule formation were stained with Alizarin red S dye, which binds to Ca^2+^ ions to stain the calcified nodule bright red. Alizarin red S staining showed that Mg ion treatment increased Ca^2+^ accumulation in extracellular matrix. We observed that 10, 50, 100, and 200 μg/L IGF‐1 increased the amount of matrix Ca^2+^ deposits after 14 days of incubation in MC3T3‐E1 cells. Among them, IGF‐1 mineralized nodules with a concentration of 200 μg/L were more significant (Figure 2).

### 3.3 Effect of IGF‐1 concentration on proliferation of MC3T3‐E1 cells

Different concentrations of IGF‐1 had a significant effect on the proliferation of MC3T3‐E1 cells, especially at a concentration of 200 μg/L. This indicated that when the concentration of IGF‐1 was 10–200 μg/L, it could significantly promote the proliferation of cells, whereas 200 μg/L IGF‐1 promoted cell proliferation (Table1).

**Table 1:** Effect of IGF‐1 concentration on proliferation of MC3T3‐E1 cells (OD value at 450 nm, mean ± *SD*, *n* = 6)

Group	Cultured time (day)			
	1	3	5	7
Control	0.38 ± 0.06	0.72 ± 0.08	1.12 ± 0.05	1.52 ± 0.05
10 μg/L	0.42 ± 0.05	0.76 ± 0.11	1.22 ± 0.06	1.56 ± 0.04
50 μg/L	0.45 ± 0.07	0.82 ± 0.07	1.29 ± 0.08	1.66 ± 0.08
100 μg/L	0.55 ± 0.04	0.96 ± 0.11	1.32 ± 0.07	1.76 ± 0.04
200 μg/L	0.67 ± 0.09	0.99 ± 0.08	1.44 ± 0.06	1.82 ± 0.08

Abbreviation: IGF, insulin‐like growth factor‐1.

### 3.4 Microarray analysis and GO analysis of IGF‐1 induced differential gene expression

**Figure 3:**
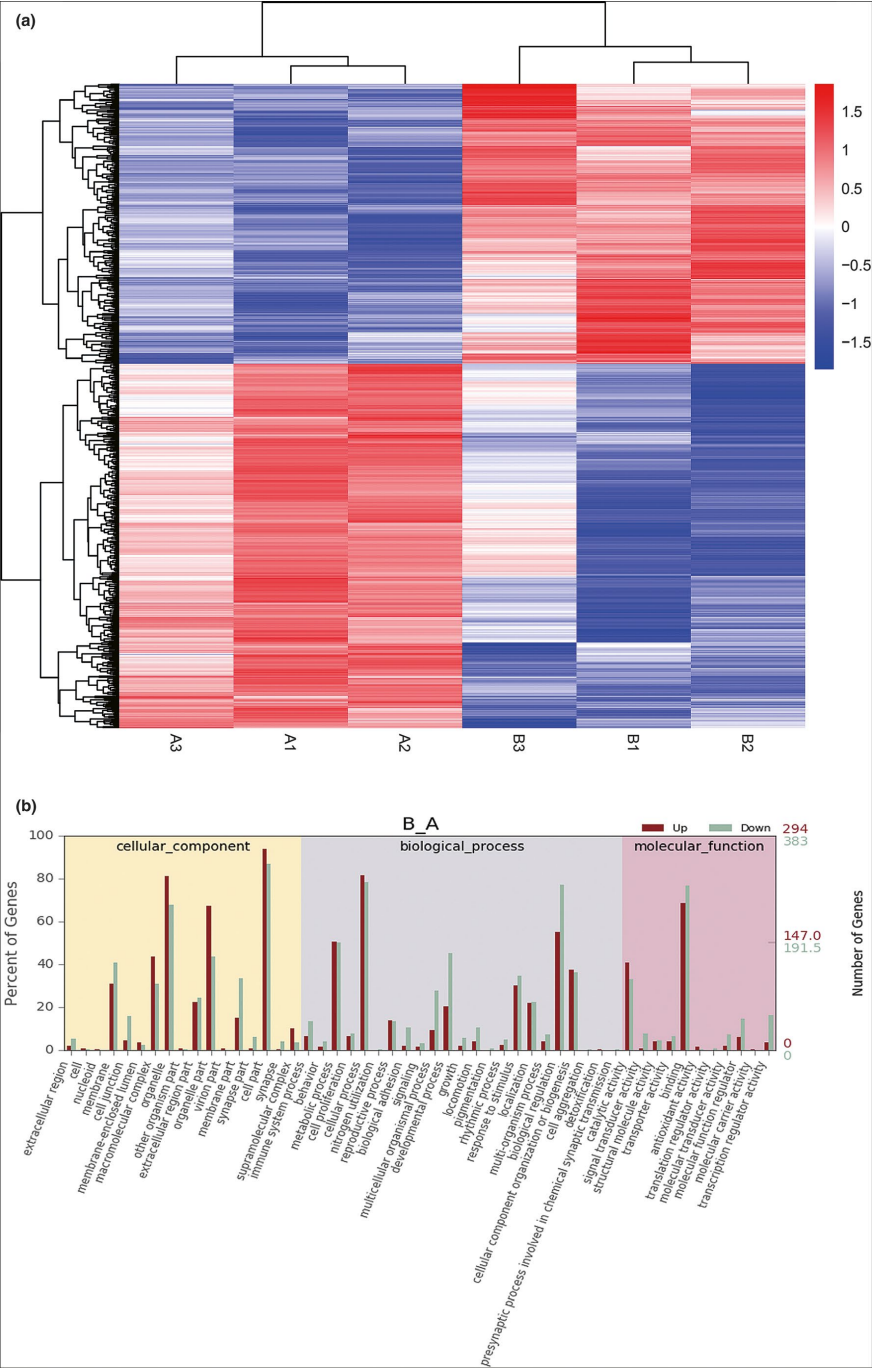
Microarray analysis and GO analysis of IGF‐1‐induced differential gene expression. (a) Clustering histogram. Compared with the control group, IGF triggered 677 differentially expressed genes (DEGs; 383 downregulated genes and 294 upregulated genes. (b) GO statistical histogram. The left ordinate is the ratio of upregulated/downregulated DEGs, the right ordinate is the number of DEGs upregulated or downregulated. DEGs, differentially expressed genes; GO, gene ontology; IGF, insulin‐like growth factor‐1

From the microarray analysis, we obtained the genome‐wide transcription of MC3T3‐E1 cells that were induced by IGF at the concentration of 200 ng/ml (Figure 3a). Compared with the control groups, 677 differentially expressed genes (DEGs) based on nominal *p* values were triggered by IGF; 383 genes were downregulated and 294 genes were upregulated. The top differentially expressed upregulated and downregulated genes between the IGF and control groups are listed in Figure 3a, respectively. A treatment‐independent clustering histogram based on 677 genes revealed the expression profiles of these genes among these groups. This heatmap indicated that these DEGs had similar expression patterns within the groups while having obviously different expression patterns between groups. GO analysis of differential gene expression induced by IGF (Figure 3b). We identified the relationship between DEGs and their main functions can through GO analysis, which included molecular function, cellular component, and biological processes, After correcting the calculated *p* value, we used *q* < 0.05 as the threshold. We defined GO items satisfying this condition as GO entries that are significantly enriched in the differentially expressed genes. Small FDR indicates an enriched GO term. As a result, we obtained a GO statistical histogram of differentially expressed genes. The abscissa is a secondary GO entry with DEGs annotation results. The left ordinate is the ratio of upregulated/downregulated DEGs, and the right ordinate is the number of DEGs upregulated or downregulated.

### 3.5 Pathway analysis of differential gene expression induced by IGF

**Figure 4:**
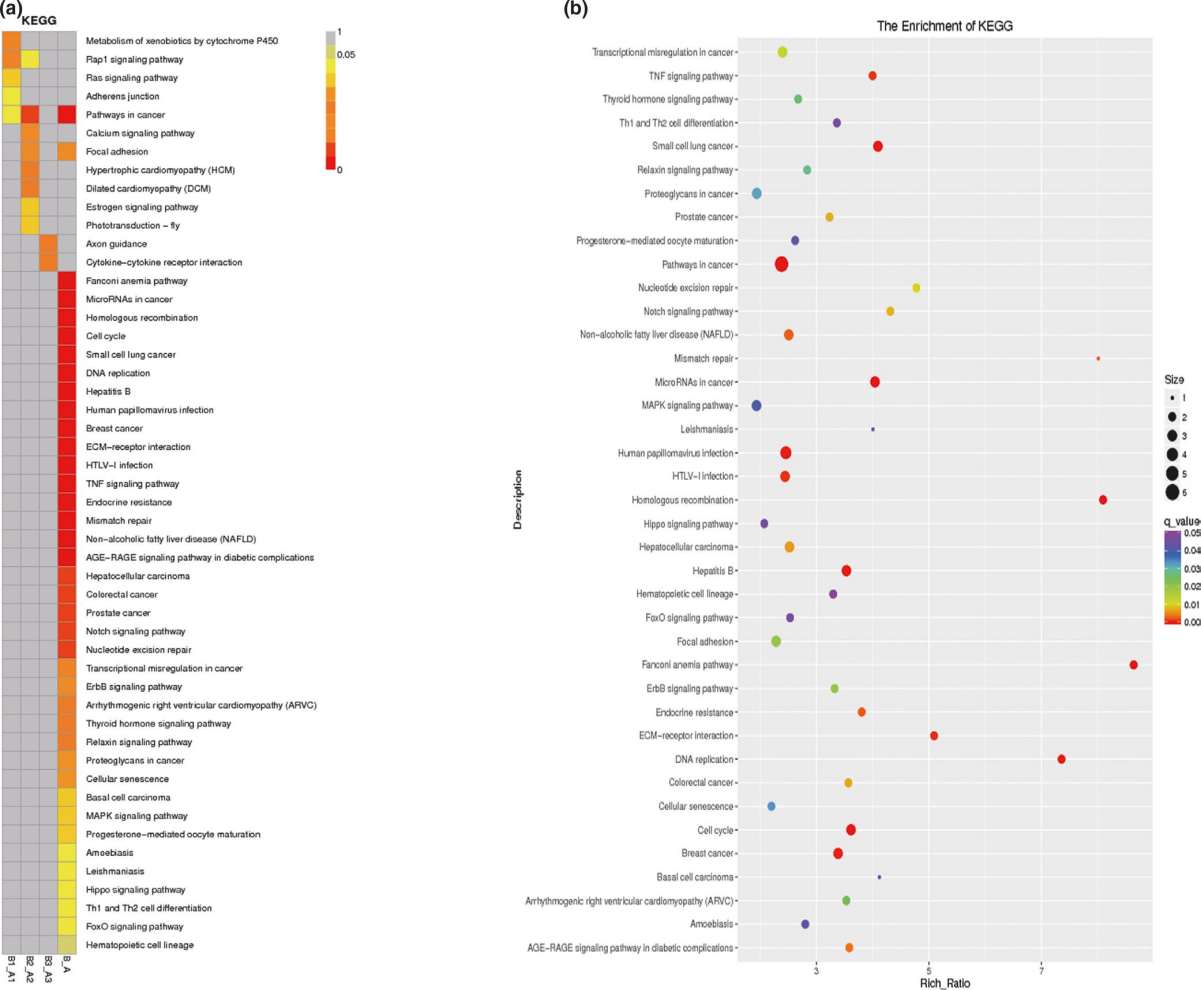
Pathway analysis of differential gene expression induced by IGF. (a) 280 important pathways in the KEGG database. (b) KEGG enrichment *q* value result graph for a single group showed 39 significant enriched pathways, including the metabolic pathways, cell cycle, TNF signaling pathway, Notch signaling pathway, and MAPK signaling pathway. IGF, insulin‐like growth factor‐1; KEGG, Kyoto Encyclopedia of Genes and Genomes; TNF, tumor necrosis factor

Considering the interactions and functions of DEGs, we recognized the significant pathways based on the Kyoto Encyclopedia of Genes and Genomes (KEGG) database. We tested a total of 280 pathways. As shown in Figure 4, we identified 39 significant enriched pathways, including the metabolic pathways, cell cycle, tumor necrosis factor (TNF) signaling pathway, Notch signaling pathway, and MAPK signaling pathway.

### 3.6 Quantitative real‐time polymerase chain reaction (qRT‐PCR) analysis and western blot analysis

**Figure 5:**
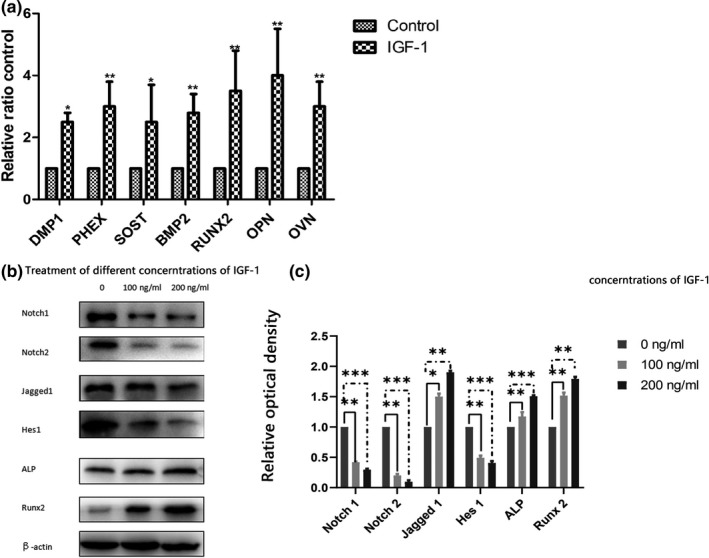
qRT‐PCR analysis and western blot analysis. (a) Gene expressions by qRT‐PCR analysis. Data are expressed as means ± *SD* from three biological repeats and three technical repeats. Compared with the control group, the expression of DMP1, PHEX, SOST, BMP2, RUNX2, OPN, and OCN increased significantly. (b) Western blot analysis of the effect of IGF‐1 on the expression of Notch signaling pathway‐related protein. (c) The density of each band was quantified with ImageJ. Our data showed that IGF‐1 downregulated the expression and activation of Notch1, Notch2, and Hes1. In contrast, the expression of Jagged1, ALP, and Runx‐2 was upregulated. Data are presented as mean ± *SEM*. *p < .05; **p < .01; ***p < .001. ALP, alkaline phosphatase; IGF, insulin‐like growth factor‐1; qRT‐PCR, quantitative real‐time polymerase chain reaction

On the basis of pathway analysis and signal‐net analysis, we selected osteogenic‐related genes (*DMP1* [RefSeq:NC_000071.6], *PHEX* [RefSeq:NC_000086.7]*, SOST* [RefSeq:NC_000077.6]*, BMP2* [RefSeq:NC_000068.7]*, RUNX2* [RefSeq:NC_000083.6]*, OPN* [RefSeq:NC_000071.6]*, OCN* [RefSeq:NC_000069.6]) that were significantly upregulated both in GO analysis and in pathway analysis to perform qRT‐PCR. As shown in Figure 5a, all the expressions of these genes were significantly increased in MC3T3‐E1 cells treated with IGF compared with the control. Fortunately, the tendencies in the qRT‐PCR results were consistent with those in the microarray analysis. Our previous data demonstrated that the Notch pathway was highly upregulated in MC3T3‐E1 cells. Therefore, to examine whether IGF‐1 plays a role in the Notch pathway, MC3T3‐E1 cells were treated with IGF‐1, and analyzed the expression and activation of Notch1, Notch2, Jagged1, Hes1, ALP, and Runx‐2 by western blot (Figure 5b). Our data showed that IGF downregulated the expression and activation of these signaling molecules (Figure 5c).

## 4 DISCUSSION

Osteoblasts are important functional cells in bone formation and bone remodeling. The proliferation and differentiation of osteoblasts are regulated by various factors. Osteoblasts are the main executive cells required for bone development, growth, and remodeling, They secrete collagen and other matrix proteins around the bones and have the function of promoting matrix calcification (Chau, Leong, & Li, 2009). As one of the most abundant growth factors in bone, IGF‐I regulates the function of osteoblasts in the form of autocrine and paracrine, and it also plays an important role in bone metabolism (Ogata and Kawaguchi, 2008). IGF‐1 can promote bone regeneration by inducing the proliferation of mouse osteoblasts to ensure the number of osteoblasts involved in bone remodelling (Guan, Ge, Liu, Ma, and Cui, 2009). IGF is one of the growth factors in bone matrix, and its protective effect on bone mass has attracted significant attention (Ueland et al., 2010). Current research indicates that IGF‐1 deficiency can reduce the likelihood of bone formation, decrease bone density, and increase the risk of fracture (Kaur et al., 2010; Yao et al., 2008). In vitro studies have found that IGF‐I significantly promotes the activity of ALP in bovine osteoblasts (Li, Yin, Guo, Zhou, and Li, 2009). In vivo experiments showed that IGF‐I promoted the synthesis of ALP and calcification of bone. Sakata et al., (2010) found that injection of IGF‐I into rats significantly increased the activity of ALP in blood and promoted bone formation and calcification of the bone matrix. However, IGF‐I promotes calcification of bone matrix. In addition to increasing ALP synthesis, other mechanisms may need further study. Osteoporosis and osteopenia caused by various causes often are accompanied by a decrease in the number of osteoblasts and a decrease in function (Fini et al., 2010). Promoting the proliferation and differentiation of osteoblasts can provide new ideas for the research and development of bone tissue anabolic drugs (Ohta, Yamada, Matuzaka, & Inoue, 2010). ALP is a specific marker for early osteoblast differentiation and often is used as an indicator of osteoblast function (Collette et al., 2010). In vitro experiments have confirmed that calcification does not occur without the presence of ALP (Chen, O'Neill, Chen, and Moe, 2010). Our results showed that IGF‐1 can promote the expression of ALP in the cytoplasm of mouse osteoblasts, and showed a dose‐dependent effect. This result indicated that IGF‐1 can promote its differentiation and maturation while also promoting cell proliferation (Guan et al., 2009).

This was the first attempt to perform a genome‐wide transcriptional analysis for a comprehensive understanding of IGF‐induced osteoblast differentiation in MC3T3‐E1 cells. On the basis of current annotation via GO analysis, we connected DEGs with potential biological pathways involved in IGF‐induced osteoblast differentiation in MC3T3‐E1 cells. As shown in Figures 3 and 4, our study revealed the significant changed up‐or downregulation of DEGs of MC3T3‐E1 cells by IGF. We implemented the GO enrichment of DEGs by the hypergeometric test, in which we calculated the *p* value and adjusted it as *q* value, and then determined the data background of the genes in the whole genome. We considered GO terms with *q* < 0.05 to be significantly enriched. GO enrichment analysis exhibited the biological functions of the DEGs. GO included molecular function, cellular components, and biological processes. Fortunately, the tendencies of the qRT‐PCR results were consistent with that of the microarray analysis. Consequently, these data indicate that these pathways were activated by IGF in MC3T3‐E1 cells. Our results revealed 39 significant enriched pathways, including the metabolic pathways, cell cycle, TNF signaling pathway, Notch signaling pathway, and MAPK signaling pathway.

The mechanism by which Notch inhibits osteogenic differentiation through Wnt signaling is thought to be accomplished by regulation of β‐catenin. In a study by Engin et al., (2008), the effect of Notch on osteoblast proliferation was also observed. This effect is thought to be involved in the increased expression of cyclin D1 and cyclin E. Hilton et al., (2008) concluded that Notch signaling seemed to inhibit osteoblast differentiation through Hes or Hey proteins, which diminished Runx2 transcriptional activity via physical interaction. These results support a model in which Notch signaling in bone marrow normally acts to maintain a pool of mesenchymal progenitors by suppressing osteoblast differentiation. The literature has indicated that inhibition of Notch1 gene expression in early osteoblasts is formed by MC3T3‐E1 cells, which causes a decrease in bone mass and can reduce the proliferation and differentiation of osteoblasts. Studies have shown that by improving Notch1 the inhibition of osteoblast differentiation caused by the expression level occurs through the inhibition of Wntβ catenin signaling, In this experiment, IGF promoted the osteogenesis of MC3T3‐E1 cells, and the expression of Notch1 was decreased (Wang, 2011). Jagged1 knockout mice died early in the embryo before bone formation because Jagged1 and Deltal deletions can damage constitutional formation and blood vessel formation. We detected the presence of Jagged1 in both osteoblasts cultured in vivo and in vitro, as well as during bone formation. In this experiment, IGF promoted the osteogenesis of MC3T3‐E1 cells, and the expression of Jagged1 was increased (Calvi et al., 2003; Nobta et al., 2005). The Hes protein usually acted as a transcriptional inhibitor; Hesl inhibited osteocalcin expression and induced transcription of osteopontin. Hesl was able to inhibit the differentiation of preadipocytes during the precursor phase, but Hesl was necessary during the late differentiation. Hesl was a downstream product of the Notch signaling pathway, and elevated Hes1 expression levels indicated that the Notch signaling pathway was activated (Kageyama, Ohtsuka, & Kobayashi, 2007; Ross, Rao, & Kadesch, 2004). Osteoblasts promoted osteoclast proliferation and differentiation via RANKL/OPG, MCSF RANKL, OPG, and MCSF are key factors regulating bone resorption and act directly on osteoclast somatic cells Schoppet, Preissner, & Hofbauer, 2002).

Alkaline phosphatase positive is a hallmark gene of osteoblasts, which is induced by osteoblast induction medium. Osteoblasts can be seen with ALP‐ positive features. We induced MC3T3‐E1 cells by IGF‐1 for 14 days. The obvious ALP‐positive staining characteristics indicated that MC3T3‐E1 cells had been successfully differentiated into bone cells (Piattelli et al., 2002). Studies have shown that the loss of Runx2 leads to the inability of osteoblast precursor cells to differentiate into mature osteoblasts such that intramembranous and extramembranous osteogenesis is completely inhibited. Transgenic mice overexpressing Runx2 in osteoblasts exhibited symptoms of osteopenia. Runx2 was able to induce ALP activity in immature bone marrow MSCs and osteoblasts in vivo and was able to increase mineral precipitation in osteoblasts. During the process of differentiation, Runx2 inhibited the Notch‐C‐RBP‐K transcriptional complex by interacting with Notchl‐IC to inhibit the Notch signaling pathway (Banerjee et al., 2015; Ducy, Zhang, Geoffroy, Ridall, & Karsenty, 1997).

As a growth factor with good application prospects, the  signal pathway for IGF1 action can be used in the treatment of osteoporosis and osteopenia, Future studies should observe the molecular mechanism of its influence on osteogenesis regulation. In‐depth research is necessary to verify whether it has clinical application value through animal experiments.

## CONFLICT OF INTEREST

None.
